# A Clinical, Pathological, Epidemiological and Molecular Investigation of Recent Outbreaks of Peste des Petits Ruminants Virus in Domestic and Wild Small Ruminants in the Abu Dhabi Emirate, United Arab Emirates

**DOI:** 10.3390/vetsci10010056

**Published:** 2023-01-13

**Authors:** Hassan Zackaria Ali Ishag, Abdelnasir Mohammed Adam Terab, Yassir Mohammed Eltahir, El Tigani Ahmed El Tigani-Asil, Nasereldien Altaib Hussein Khalil, Esamt Faisal Malik Gasim, Mohd Farouk Yuosf, Saeed Mohamed Saeed Al Yammahi, Asma Mohammed Amer Al Mansoori, Salama Suhail Mohammed Al Muhairi, Zulaikha Mohamed Abdel Hameed Al Hammadi, Asma Abdi Mohamed Shah, Majd Mohamed Azmi Naser Alherbawi, Mervat Mari Hassan Al Nuaimat, Oum Keltoum Bensalah, Abdelmalik Ibrahim Khalafalla

**Affiliations:** 1 Veterinary Laboratories Division, Animal Wealth Sector, Abu Dhabi Agriculture and Food Safety Authority (ADAFSA), Abu Dhabi P.O. Box 52150, United Arab Emirates; 2 Animal Health Division, Animal Wealth Sector, Abu Dhabi Agriculture and Food Safety Authority (ADAFSA), Abu Dhabi P.O. Box 52150, United Arab Emirates; 3 National Emergency Crisis and Disasters Management Authority (NCEMA), Abu Dhabi P.O. Box 113811, United Arab Emirates; 4 Animal Development and Health, Ministry of Climate Change & Environment, Dubai P.O. Box 1509, United Arab Emirates

**Keywords:** peste des petits ruminants, PPRV, goats, dama gazelle, lineage IV, UAE

## Abstract

**Simple Summary:**

Peste des petits ruminants (PPR) is a contagious disease in domestic small ruminants. The virus can also infect wildlife, with unknown roles in PPR epidemiology. In the United Arab Emirates (UAE), the previously published reports were from wildlife only. This is a first report from the UAE describing PPRV in domestic small ruminants. From four outbreak notifications, the disease is investigated by clinical, pathological, and molecular studies. We found that the clinical and pathological forms of the disease were almost identical in all examined animals and compliant to the classical forms of the disease. Phylogenetic analysis based on the N gene and F gene classified the virus within Asian lineage IV.

**Abstract:**

(1) Background: Peste des petits ruminants (PPR) is a highly contagious animal disease affecting small ruminants, leading to significant economic losses. There has been little published data on PPR virus (PPRV) infection in the United Arab Emirates (UAE); (2) Methods: four outbreaks reported in goats and Dama gazelle in 2021 were investigated using pathological and molecular testing; (3) Results: The infected animals showed symptoms of dyspnea, oculo-nasal secretions, cough, and diarrhea. Necropsy findings were almost similar in all examined animals and compliant to the classical forms of the disease. Phylogenetic analysis based on N gene and F gene partial sequences revealed a circulation of PPRV Asian lineage IV in the UAE, and these sequences clustered close to the sequences of PPRV from United Arab Emirates, Pakistan, Tajikistan and Iran; (4) Conclusions: PPRV Asian lineage IV is currently circulating in the UAE. To the best of our knowledge, this is a first study describing PPRV in domestic small ruminant in the UAE.

## 1. Introduction

The peste des petits ruminants (PPR), as a disease, was first described in West Africa in 1942 [1]. It is economically significant, causing death of small ruminants and affecting the lives of over 330 million low-income livestock keepers in Africa, the Middle East, and Asia [2]. As a result, in order to completely eliminate PPR by the year 2030, the Food and Agriculture Organization (FAO) and the World Organization for Animal Health (WOAH) launched the PPR Global Control and Eradication Strategy (PPR GCES) in 2015 [2].

Peste des petits ruminants virus (PPRV), is classified as a member of the family *Paramyxoviridae*, of the genus *Morbillivirus*, of the species *Small Ruminant Morbillivirus* by the International Committee on Taxonomy of Viruses (ICTV) [3], is the cause of the disease. PPRV is an enveloped virus with a single-stranded negative-sense RNA genome of approximately 16 kb in length that encodes six structural proteins: the nucleocapsid protein (N), the phosphoprotein (P), the matrix protein (M), the fusion protein (F), the haemagglutinin protein (H), the large polymerase protein (L), and two non-structural proteins (C and V), encoded in alternative open reading frames in the P gene [4,5,6]. The virus only has one serotype, but using phylogenetic analysis of the fusion (F) protein gene and nucleocapsid protein (N) gene, the virus can be classified into four lineages (I-IV) [7,8]. The geographical spread of the virus is reflected in the PPRV lineages, with lineages I and II being isolated only from West African nations. Lineage III is found in the Arabian Peninsula (Yemen, Qatar, and Oman) and East Africa, while Lineage IV is only found in the Middle East, with some extending into southern India, southern Asia, Turkey, and African territories [8,9,10]. However, recent studies have reported lineage II’s emergence in China [11]. PPRV can infect domestics, wild small ruminants [12] and camels [13,14]. Sheep and goats are considered the natural hosts of PPRV [15]. However, PPRV has been diagnosed in wild small ruminants, including *Gazellinae* (Dorcas gazelle), *Caprinae* (Nubian ibex and Laristan sheep), *Hippotraginae* (gemsbok), and *Capra aegagrus blythi* (Sindh Ibex) in several countries including Pakistan and the UAE [16,17,18]. Nevertheless, wildlife’s role in the epidemiology of PPR is still unclear.

The virus is transmitted through direct contact between infected and susceptible animals. The presence of mixed populations (i.e., flock of sheep and goats), the introduction of new animals into a flock/herd and uncontrolled cross-border animal movement are major risk factors for the spread of PPR [19,20]. 

Depending on the risk factors and the virulence of the virus that is infecting the animal, the disease manifests in the animal in per-acute, acute, sub-acute, and subclinical forms. However, PPR is commonly acute in sheep and goats [21]. The gross pathological changes of PPR in sheep and goats are mainly in the digestive system, which include ulcerative to necrotic lesions on the buccal cavity (dental pad, gum, dorsal surface of the tongue, palatine tonsil, and hard palate), as well as congestion, hyperemia, edema of the intestines and hemorrhagic colitis [7,21]. Gross lesions also occur in the respiratory system including frothy exudates in the trachea, fibrino-necrotic tracheitis, bronchitis, consolidation with alternative atelectasis-emphysema, and dark-red discoloration of the cranial pulmonary lobes with fibrin depositions on the pleural surface [22,23]. Congestion and bronchopneumonia are associated with bacterial infection [24]. Similar lesions to that of sheep and goats were reported in wild small ruminants except for oral mucosa lesions, which were not observed [18]. Histopathological changes of PPRV-infected tissues include syncytial cell formation, particularly in the oral mucosa, lung alveoli, liver, and lymphoid tissues with extensive necrosis. In the lung, alveolar macrophages with intranuclear and intracytoplasmic inclusion bodies, lymphocytes, numerous neutrophils, fibrin exudates, and multinucleated giant cells have also been reported in severe infection although some of these histopathological changes may result from secondary bacterial or parasitic infections [7,22]. 

Current diagnostic methods for suspected PPR cases include clinical, pathological examinations and laboratory confirmation using molecular tests such as real-time quantitative PCR (RT-qPCR) and traditional reverse transcription polymerase chain reaction (RT-PCR). The viral lineage is typically determined by sequencing a portion of the nucleocapsid or the fusion protein genes or by whole genome sequencing [25].

Globally, PPR is endemic in huge parts of the sub-Saharan Africa, the Arabian Peninsula, the Middle East and Asia [26,27,28,29,30]. In the UAE, PPR caused by the lineage III virus was first reported in wildlife in 1986 [17]. Another report also described PPRV in Arabian wildlife in the UAE in 2009, which was caused by the Asian lineage IV virus [18]. In 2022, another study from experimental infection of goats with PPRV originated from Barbary Sheep in the UAE was also described the virus within Asian lineage IV [25]. Since 2005, a total of 62 outbreaks of PPR in small ruminants and wildlife species in the UAE have been reported to the WOAH [31,32]. Despite this, no lineage information for the PPRV in domestic animals in the UAE is available. Additionally, the host range of the PPR virus has expanded to include large ruminants (camels, cattle, and water buffalo) and wild small animals over the past few decades, raising concerns about the ongoing PPR global eradication program, which is primarily focused on the reachable sheep and goat populations [33]. Therefore, the present study aims to characterize PPRV strains currently circulating in the domestic and wild small ruminants in the UAE, and to study the clinical and pathological features of the disease during the response to PPR outbreak notifications within Abu Dhabi Emirate in 2021. The present dominant PPRV lineage in the UAE was further defined based on sequencing and phylogenetic analysis of the F gene and N gene, and its risk factors for occurrence were reviewed.

## 2. Materials and Methods

### 2.1. Epidemiological Data, Necropsy and Sampling

**Figure 1:**
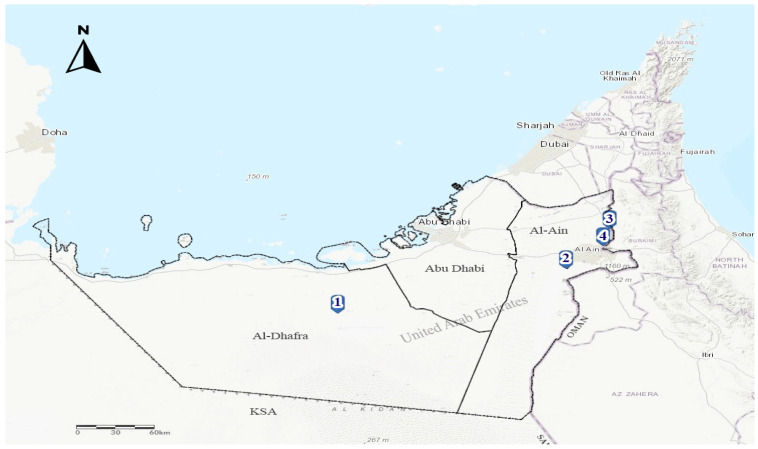
Map showing the location of the four PPRV infected farms in Al Ain region (Farms 2, 3 and 4) and Al Dhafra region (Farm 1), Abu Dhabi Emirate, UAE.

In response to four PPR outbreaks notifications that occurred in Abu Dhabi Emirate in July–September 2021, PPR suspected goats (*n* = 3) and dama gazelle (*n* = 1) (Figure 1) with a history of fever, dyspnoea, oculo-nasal mucopurulent discharges, cough, and diarrhea were received at the Abu Dhabi Agriculture and Food Safety Authority (ADAFSA) veterinary laboratories for necropsy examination and laboratory diagnosis.

Animals originated from four different breeding farms located in two regions, Al Dhafra (Farm 1) and Al Ain (Farms 2, 3, and 4) within Abu Dhabi Emirate (Figure 1). Farms (1, 3, and 4) contains sheep, goats, whereas farm 2 contains dama gazelle species. Sheep and goats present in the three farms are from local breeds and were raised for breeding purposes. The origin of the dama gazelle species was unknown (Table 1).

Gross lesions were recorded after necropsy examinations, lung and intestine samples were collected for histopathology. In addition, a total of 10 samples, including spleen (*n* = 3), nasal swabs (*n* = 2), oral swabs (*n* = 2), lung (*n* = 1), intestine (*n* = 1), and lymph node (*n* = 1), were taken from three goats and one dama gazelle (Table 1) and shipped on ice to the ADAFSA veterinary laboratories for investigation.

### 2.2. Histopathological Examination

Tissue samples, including intestine and lung from one goat, were fixed in 10% neutral formalin for 24–48 h at room temperature for histopathological examinations following a previously described method [34]. Formalin-fixed tissue samples were processed in an automatic tissue processor (ATP1-220, Triangle Biomedical Sciences, Durham, NC, USA), embedded in paraffin blocks, and cut into 5 µm thick sections. For microscopic examination, sections were stained with Hematoxylin and Eosin (H&E) (Thermo Fisher Scientific, Runcorn, Cheshire, UK). Images were captured using the VisionTek digital microscopy system (DM01, Sakura Finetek, Torrance, CA, USA).

### 2.3. Quantitative Real-Time PCR (RT-qPCR) Based Detection of PPRV

Total RNA extraction from each biological sample was performed using the EZ1 Virus Mini Kit V2.0 (48) (Qiagen, Hilden, Germany) as per the kit instructions. Briefly, 60 µL of the RNA was eluted from 400 µL of the swab or tissue lysate using the Advanced EZ1 instrument (Qiagen in Hilden, Germany). The presence of PPRV was detected and confirmed by real-time quantitative polymerase chain reaction (RT-qPCR) targeting the N gene of PPRV as previously described [35]. The primer sets used were PPRNF forward primer (5′-CACAGCAGAGGAAGCCAAACT-3′), PPRNR reverse primer (5′-TGTTTTGTGCTGGAGGAAGGA-3′), and the PPRNP TaqMan probe (FAM-5′-CTCG-GAAATCGCCTCGCAGGCT-3′-BHQ1). The reaction mixture in a total volume of 20 μL, consisted of 12 μL of Master Mix (Roche, Basel, Switzerland), 5 μL tested RNA and 1 μL of each primer (10 pmol/µL) and probe solution (10 pmol/µL), using the Real-time ready RNA Virus Master Kit (Roche). The following thermal profile was applied: an initial reverse transcription at 45 °C for 30 min, followed by reverse transcriptase inactivation and DNA polymerase activation at 95 °C for 5 min and 50 cycles of amplification (15 s at 95 °C and 30 s at 60 °C). The RT-qPCR analysis was performed using the LightCycler^®^ 2.0 Instrument (Roche, Life Science, Basel, Switzerland). 

### 2.4. N Gene and F Gene Sequencing and Phylogenetic Analysis

#### 2.4.1. Amplification of N Gene and F Gene

The QIAGEN OneStep RT-PCR Kit (QIAGEN, Germany) was used to amplify N gene and F gene from a representative sample from each outbreak (shown in bold in Table 1) following the manufacturer’s instructions. The N gene (351 bp) was amplified with NP3: 5′-GTCTCGGAAATCGCCTCACAGACT-3′ and NP4: 5′-CCTCCTCCTGGTCCTCCAGAATCT-3′. The PCR thermal profile was set to 95 °C for 10 min for the initial denaturation and polymerase activation, followed by 35 cycles of 94 °C for 30 s, 55 °C for 30 s and 72 °C for 1 min. Amplification of the F gene (448 bp) was carried out using the primers PPRV-F: (5′-AGTACAAAAGATTGCTGATCACAGT-3′) and PPRV-R: (5′-GGGTCTCGAAGGCTAGGCCCGAATA-3′) [36]. The PCR thermal profile was set to 50 °C for 30 min for reverse transcription, 95 °C for 15 min for enzyme inactivation, followed by 95 °C for activation of the polymerase followed by 35 cycles of 94 °C for 1 min, 50 °C for 1 min and 72 °C for 2 min. The final extension was conducted at 72 °C for 7 min. The amplicon was visualized in a 1.8% agarose gel. 

#### 2.4.2. Sanger Sequencing

ExoSAP-ITTM PCR Product Cleanup Reagent (Thermo Fisher Scientific, Waltham, MA, USA) was used to purify the PCR products of the N gene and F gene, and BigDye Terminator v3.1 Cycle Sequencing kit (Applied Biosystems, Waltham, MA, USA) was utilized to perform bidirectional Sanger sequencing using the same primers used to amplify N gene and F gene and BigDye Terminator v3.1 Cycle Sequencing kit (Applied Biosystems, Waltham, MA, USA) as previously described [36]. The 20 μL reactions consisted of 9 μL of water, 3.5 μL of 5× Sequencing Buffer, 1 μL of the BigDye Terminator v3.1, 1 μL of 3.2 pmol of each primer, and 5.5 μL of DNA. The BigDye XTerminator^TM^ Purification kit (Applied Biosystems) was used to purify the reaction mixture in accordance with the manufacturer’s instructions. Sequencing was performed on a SeqStudio Genetic Analyzer (Applied Biosystems, Waltham, MA, USA) using the ‘MediumSeq BDX’ run module. The sequence trimming and assembly were performed with CLC Genomic Workbench v.20 (Qiagen, Aarhus, Denmark). The consensus sequences of N gene and F gene were first subjected to NCBI BLAST analysis. These sequences were also deposited in the GenBank database under accession numbers shown in Table 1.

#### 2.4.3. Sequence Alignment and Phylogenetic Analysis

Multiple sequence alignments of 255 bp and 322 bp of the N gene and F gene of the PPRV UAE strains characterized in this study were performed with the ClustalW program [37] impeded in MEGA X. Each sequence of the N gene or F gene was aligned with their corresponding PPRV curated sequences recently published [6] which are available in the PPR Network online page (https://www.ppr-labs-oie-network.org/materials-and-protocols/pprv-sequence-datasets, accessed on 13 December 2022). A newly published sequence from the UAE wildlife (GenBank: OM867572.1) [25] is also included in the phylogenetic tree. The MEGA X program [38] was used to construct the phylogenetic tree using the Maximum Likelihood method and the General Time Reversible model [39], with 1000 Bootstrap confidence.

## 3. Results

### 3.1. Outbreak Investigations

Four PPR outbreak notifications were received by ADAFSA epidemiology unit during the period July–September 2021 from Al Ain (3 outbreaks) and Al Dhafra (one outbreak) regions in the Abu Dhabi Emirate (Figure 1) and (Table 2). Upon outbreaks investigations, animal species affected included goats in three mixed (with sheep) breeding farms and a dama gazelle in one farm. The latter is located within Al Ain region, where high (60%) intensity of small ruminant farms is present. The age of the infected goats varies from less than three months to 1 year old, whereas the infected gazelles were around 1–2 years old. Two infected goats (farms 1 and 3) together with the dama gazelles (farm 2) were unvaccinated against PPR. In contrast, one goat infected (farm 4) was vaccinated seven months before the outbreak. The infected (farms 1 and 3) also had a recent history (3 weeks before the outbreak) of introducing new unvaccinated animals. These were from a farm within Abu Dhabi emirate (farm 1) and from another emirate regarding farm 3.

The morbidity rate varies from 5–9% among the infected goat herds in farms (1, 3, and 4), whereas it was 80% in the dama gazelle herd. The highest mortality and case fatality rates of 6.9% and 80% were observed in farm 3, which contains sheep and goats. In contrast, 20% and 25% of mortality and case fatality rates were reported in the infected dama gazelle in farm 2 (Table 2).

### 3.2. Clinical and Pathological Findings

**Figure 2:**
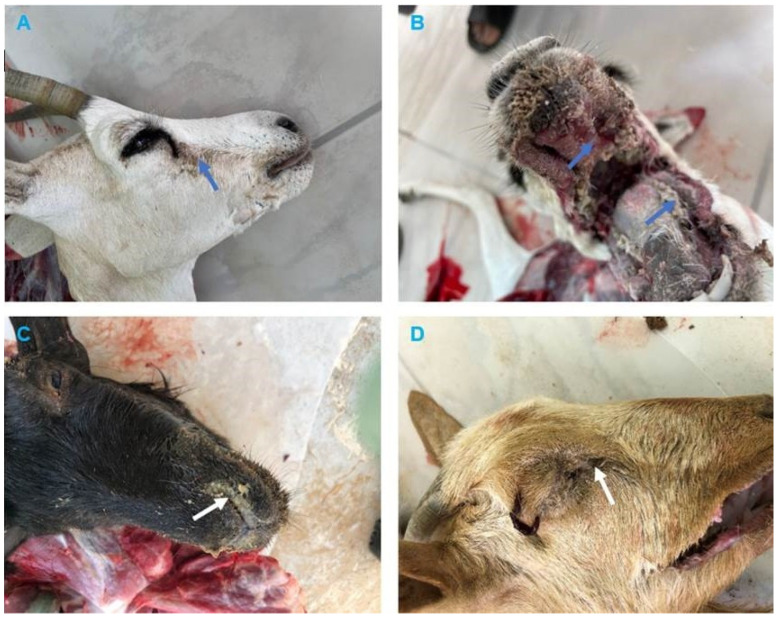
Clinical findings of peste des petits ruminants (PPR) in Dama Gazelle and Goats. (**A**) Dama Gazelle, showing ocular discharges (blue arrow). (**B**) Dama Gazelle, mouth lesions showing erosion on the buccal cavity, gums, and dental pad with whitish covering materials (blue arrows). (**C**) Goat, showing mucopurulent nasal discharges (white arrow). (**D**) Goat, showing conjunctivitis with purulent ocular discharges, the hair below the eyes matting together the eyelids (white arrow).

**Figure 3:**
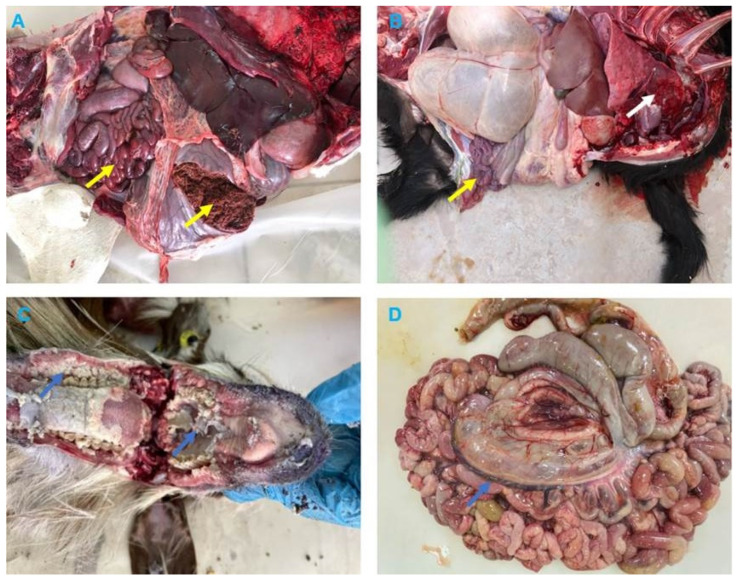
Necropsy findings in Dama Gazelle and Goats with Peste des petits ruminants (PPR). (**A**) Dama Gazelle digestive system shows hemorrhagic gastroenteritis (yellow arrows). (**B**) Goat, lung, showing bronchopneumonia, with consolidation in the middle and apical lobes of the right lung (white arrow), intestine with hemorrhagic enteritis (yellow arrow). (**C**) Goat, showing erosion on the buccal cavity, gums, and dental pad with whitish covering materials (blue arrows). (**D**) Goat, showing enlarged congested and edematous mesenteric lymph nodes (blue arrow).

The clinical signs reported by the field veterinarians for animals presented for necropsy (goats and dama gazelle) were fever, anorexia, serous-mucopurulent, oculonasal discharges, coughing, and diarrhea. At autopsy, the carcasses were fair and dehydrated. All animals exhibited erosive ulcerative lesions with fibrinous pseudo-membranes on the tongue, gum, dental pads, hard palate, and throat mucosa. Nostrils showed mucopurulent discharges and congested mucosa. The lungs showed suppurative bronchopneumonia characterized by consolidation atelectasis, red-dark discoloration of the cranioventral parts (in two goats), congestion and hemorrhage in dama gazelle. The intestine showed hemorrhagic inflammation with hypertrophy of mesenteric lymph nodes seen in all animals (Figure 2 and Figure 3). 

### 3.3. Histopathological Analysis

**Figure 4:**
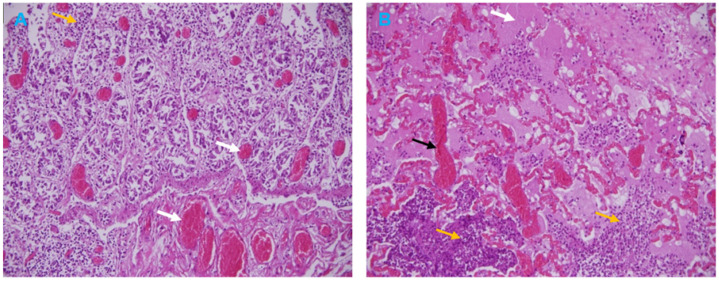
Microscopic lesions of PPR in a Goat. (**A**) Intestine section showing hemorrhagic enteritis with vascular changes (white arrows) and infiltration of inflammatory cells (yellow arrow). (**B**) Lung section showing acute hemorrhagic pneumonia with vascular changes including edema (white arrow), congestion (black arrow) and infiltration of inflammatory cells (yellow arrows). H&E stain, 20×.

Histological examination of the goat intestine section showed necrosis of the villous tips, vascular changes, and infiltration of inflammatory cells in the lamina propria (Figure 4A). The broncho-interstitial pneumonia was the main feature of the lung histological results. The lesions were edema, congestion, and hemorrhage, with infiltration of mononuclear cells in the alveoli and the alveolar septa causing thickened interalveolar septa (Figure 4B).

### 3.4. Molecular Detection and Characterization of PPRV

#### 3.4.1. Detection of PPRV by N Gene Real-Time PCR

A total of ten samples (nasal swabs = 2, oral swabs = 2, lung = 1, spleen = 3, lymph node = 1, and intestine = 1) from four different animals (3 Goats and 1 Dama Gazelle) related to four PPR outbreaks were tested positive for PPRV by RT-qPCR. The Ct value for all samples tested ranged between 20 to 25.

#### 3.4.2. Characterization of PPRV Strains and Determination of its Geographical Lineage

The BLAST results of the partial N gene and F gene of sequences obtained in this study, reveal a 99% nucleotide similarity to the PPRV strain from Barbary sheep recently isolated in the UAE (GenBank: OM867572). 

**Figure 5:**
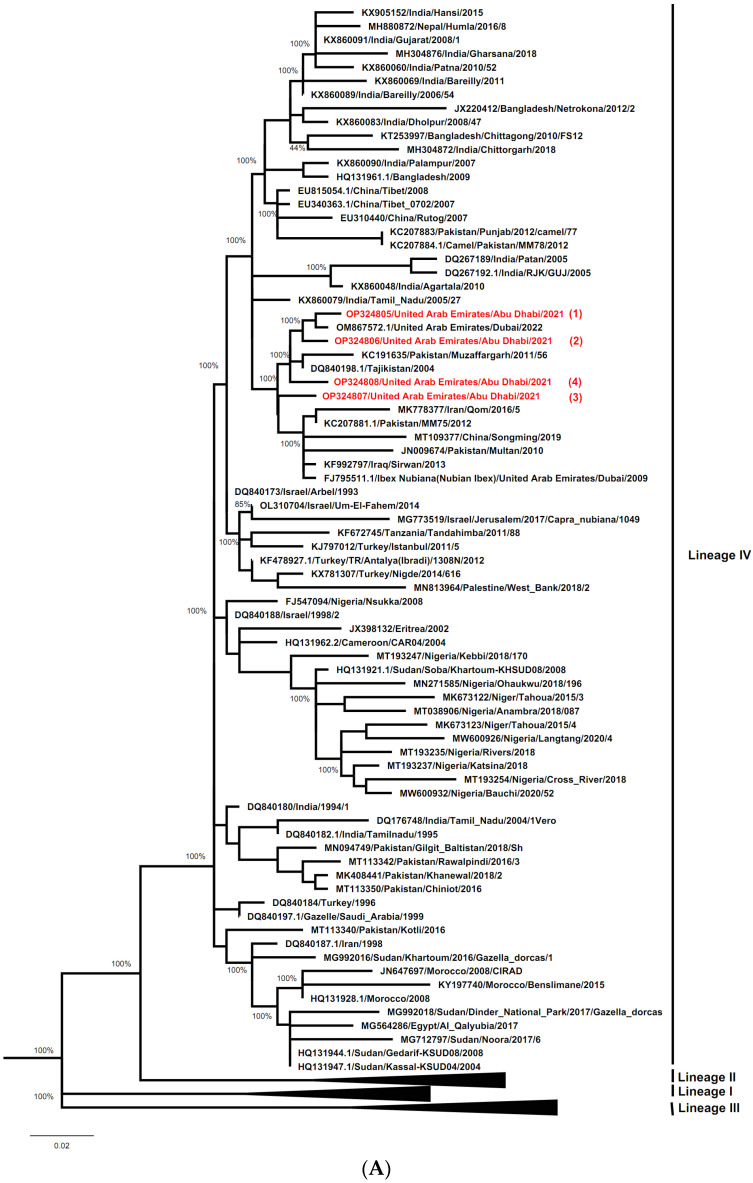

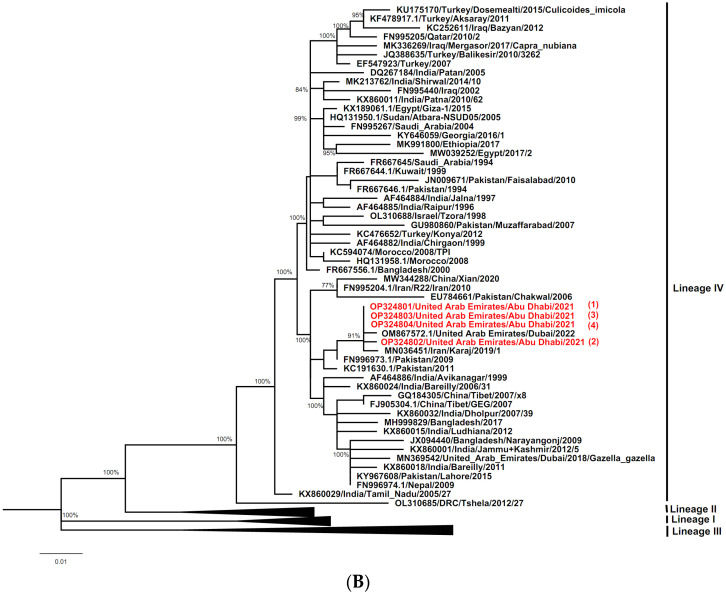
(**A**) Phylogenetic relationship between PPRV detected in the UAE in goats and dama gazelle and other virus isolates sequences described in method section. The tree was constructed based on the N gene (255 bp), using the Maximum Likelihood method and General Time Reversible model [39] in MEGAX [38]. Alignments were calculated with ClustalW [37] impeded in MEGAX. Bootstrapping (values indicated in %) was performed with 1000 replicates. The sequences obtained in this study clustered within Asian lineage IV and are marked with red color (numbered from 1–4). (**B**) Phylogenetic relationship between PPRV detected in UAE in goats and dama gazelle and other virus isolates sequences described in method section. The tree was constructed based on the F gene (322 bp), using the Maximum Likelihood method and General Time Reversible model [39] in MEGAX [38]. Alignments were calculated with ClustalW [37] impeded in MEGAX. Bootstrapping (values indicated in %) was performed with 1000 replicates. The sequences obtained in this study clustered within Asian lineage IV and are marked in red (numbered from 1–4).

Separate phylogenetic trees were constructed using either N gene or F gene sequences of the UAE PPRV strains characterized here (ADAFSA_PPRV1_2021, ADAFSA_PPRV2_2021, ADAFSA_PPRV3_2021 and ADAFSA_PPRV4_2021), and their corresponding curated sequences mentioned in the method section. All UAE-PPRV strains from goats and dama gazelle studied here, were located within the Asian Lineage IV, close to PPRV from United Arab Emirates (GenBank: OM867572.1), Pakistan (GenBank: KC191635) and Tajikistan (GenBank: DQ840198.1) based on N gene sequence analysis and close to PPRV isolated from United Arab Emirates (GenBank: OM867572.1), Iran (GenBank: MN036451) and Pakistan (GenBank: KC191630 and FN996973.1) based on F gene sequence analysis as shown in Figure 5A,B for N gene and F genes respectively.

Based on N gene sequence analysis, the UAE-PPRV strains from goats and dama gazelle obtained in this study shares identity between 97% to 98% among them and 98–99% when compared to recently sequence from Barberry sheep detected in UAE [25], shown in Table 3. 

Based on F gene sequence analysis, the UAE PPRV strains sequences are 99–100% identical, but share an identity of 98–99% with recently sequenced Barberry sheep detected in UAE [25], shown in Table 4. 

## 4. Discussion

The PPR Global Control and Eradication Strategy (GCES), released by FAO and WOAH in 2015, aims for global eradication of the disease by 2030 [2]. For a country to proceed from control to eradicate PPR, the GCES follows a technical sequential stepwise approach (stage 1 to stage 4): assessment, control, eradication, and post eradication. The PPR Monitoring and Assessment Tool (PMAT) is a companion tool to the PPR GCES to determine a country’s stage and to provide guidance to countries. It takes into consideration the five technical elements identified in the GCES (Diagnostics, Surveillance, PPR Prevention, and Control, Legal framework, and Stakeholder involvement) [40].

The UAE is currently at the eradication stage 2 of the four stages of the progressive stepwise approach for preventing and controlling PPR. To proceed to post eradication stage 4, genome information of PPRV isolates causing new outbreaks should be available [2]. Previous published reports of PPRV in 1986, 2009 and 2022 in UAE were from wildlife only. To our knowledge, the characterization of the virus in domestic small ruminant is first reported in this study.

Since PPRV recently caused epidemics in both domestic livestock and wildlife in the UAE, this research was conducted to better understand its molecular epidemiology. Four PPR outbreaks were documented in the Al Ain and Al Dhafra regions of the Abu Dhabi Emirate between July and September 2021. 

Animal species infected included goats in three farms and a dama gazelle in one farm. The age of infected goats in the three infected farms varies from less than three months to one year old. Two infected goats farms were unvaccinated and had a history of introducing new unvaccinated animals from other locations within the UAE, whereas one infected goat was from a vaccinated farm seven months before the report of the outbreak. A young age and animal movement are important risk factors for PPR occurrence [41]. The UAE national animal health plan aimed to control and eradicate PPR by 2025 follows the global PPR control and eradication strategy, which adopts mass vaccination of sheep and goats against PPR to control and eliminate the disease from the globe by 2030. In the Abu Dhabi emirate the PPR vaccine containing the Nig75/1 reference strain is used for one round of annual mass vaccination campaigns targeting adult animals (above three month of age). The program is also assessed by serological post vaccination testing with a good result of immunization coverage. Thus, the animals infected in the vaccinated farm were probably not eligible to be vaccinated (below three months) during the campaign period. Immunity against PPR in vaccinated sheep and goats is likely life-long. However, due to the high rates of population turnover, the coverage of the vaccinated animals may decline by as much as 25% yearly, or even more quickly if vulnerable animals such as newborns are added to the animal holding [42]. Therefore, it is necessary to increase vaccination coverage together with controlling animal movement between susceptible farms.

In our study, the sheep did not show clinical symptoms during the outbreak, therefore they were not examined. The susceptibility of goats to be severely affected by PPR compared to sheep is reported by many authors. Clinically, a significantly higher proportion of goats (90.9%) were reported to be sick compared to only 9.1% of sheep [43,44]. Thus, the above-mentioned report results are in line with those reported in this study.

In this study, one outbreak of PPR was reported in a dama gazelle farm, which was surrounded by a high density of domestic small ruminant farms. Available evidence indicates that PPR does not persist in wildlife populations [45]. Therefore, controlling the disease around wildlife captivities is necessary by increasing vaccination coverage of small ruminants. Moreover, monitoring the condition through surveillance, which includes sheep, goats, wild small ruminants, and a typical PPRV hosts such as camels across the UAE, will help in identifying risks of PPRV across the country. The diagnosis of PPR infection in the UAE was based on clinicopathology of the outbreaks and further confirmed by molecular methods. According to research, PPR in wild small ruminants exhibited symptoms comparable to those seen in infected sheep and goats, such as pneumonia, necrosis, and the depletion of different lymphatic tissues [46].

Search of literature revealed lack of a comprehensive clinical description of PPR in wild small ruminants such as the gazelle. In this investigation, we reported erosive mucosal lesions in the upper digestive and respiratory tracts of dama gazelle, which are typical of PPR in sheep and goats [46]. The small erosions on the tongue of sacrificed gazelles were previously described [47]. The dama gazelle in this investigation did not exhibit the lung lesions that were seen in goats (suppurative bronchopneumonia, which is characterized by consolidation of the cranioventral sections). Microscopic findings of the intestine and lung sections in goats are comparable with those reported previously [7,22].

The phylogenetic analysis based on both the N gene and the F gene classified the virus within Asian lineage IV, which is currently circulating in neighboring countries and globally [8,10]. These UAE-PPRV strains from goats and dama gazelle studied here were located within the Asian Lineage IV, close to PPRV from United Arab Emirates, Pakistan, Tajikistan and Iran for N gene and F genes sequence analysis respectively. However, the way of introduction of the virus from outside the country into UAE remains unclear. As PPRV needs close contact for transmission, these new outbreaks within UAE are likely the result of the introduction of the virus from a farm within Abu Dhabi emirate (farm 1) and from another emirate regarding farm 3. It has been concluded that two lineages (lineage III in 1986) and (lineage IV in 2009 and 2022) have been reported in UAE since 1986, but only lineage IV is currently circulating according to recently published data.

Many similarities in the F gene analysis were reported, and the clustering was more apparent in the N gene tree topology than in the F gene tree as we observed in our study. This is due to the previously proved fact that the N gene provides a better epidemiological picture of PPRV compared to the F gene classification [8,24,48,49]. However, given that PPRV tends to mutate, phylogenetic analysis based on both F gene and N gene likely provides a more informative characterization as previously described [50]. 

## 5. Conclusions

In conclusion, PPRV circulation in the UAE was significantly influenced by the age of susceptible animals under one year old and migration of unvaccinated animals. In turn, this emphasizes how crucial it is to restrict the movement of animals within the country or at international borders, boost immunization rates, and closely monitor the disease in both domestic and wild animals. Based on the phylogenetic analysis of the F gene and N gene, the PPRV strains detected in our study were classified within Asian lineage IV. The present updated information on the sequences of PPRV strains circulating in domestic and wild small ruminants in the United Arab Emirates satisfies GCES standards and advances efforts to carry out the UAE-PPR national control plan, which aims to eradicate the disease by 2025. 

## Figures and Tables

**Table 1:** Information of the samples used in the study.

Batch Number/Farm	Date of Collection	Sample Type	Animal Species	RT-qPCR Positive	GenBank Accessions	
					F Gene	N Gene
1,092,884/Farm 1	7 July 2021	**Lung**, Oral swab	Goat	2	OP324801	OP324805
221,435/Farm 2	25 July 2021	Spleen, Nasal swab, Oral swab, **Intestine**	Dama Gazelle	4	OP324802	OP324806
221,258/Farm 3	7 July 2021	Lung, Oral swab, **Lymph node**	Goat	3	OP324803	OP324807
1,093,756/Farm 4	13 September 2021	**Spleen**	Goat	1	OP324804	OP324808

NB: samples used in sequencing were in bold.

**Table 2:** The morbidity, mortality, case fatalities and vaccination history are shown. Animal species infected per each farm were shown in bold.

Farm Number	Date of Outbreak Notification	Animal Species	Total Population	No. of Sheep	No. of Goats	No. of Infected	No. of Deaths	Morbidity Rate	Mortality Rate	Case Fatality Rate	Age of Infected Animals	Vaccination Status of Infected Farm	Introduction of New Unvaccinated Animals
1	7 July 2021	Sheep and **Goats**	343	60	283	30	6	9%	1.75%	20%	<3 Months	Unvaccinated	Yes
2	25 July 2021	**Dama Gazelle**	20	0	0	16	4	80%	20.00%	25%	1–2 Years	Unvaccinated	No
3	7 July 2021	Sheep and **Goats**	232	99	133	20	16	9%	6.90%	80%	1 Year	Unvaccinated	Yes
4	13 September 2021	Sheep and **Goats**	207	79	128	10	4	5%	1.93%	40%	>6 Months	Vaccinated (11 February 2021)	No

**Table 3:** Estimation of pairwise identity between PPRV N gene of this study strains and the newly published sequence from Barberry sheep (OM867572.1/UAE/Dubai/2022). Numbers in brackets are PPRV UAE 2021 strains.

Strain	OP324805	OP324806	OP324807	OP324808	OM867572.1
OP324805/UAE/Abu Dhabi/2021 (1)		98%	98%	97%	99%
OP324806/UAE/Abu Dhabi/2021 (2)	98%		98%	98%	98%
OP324807/UAE/Abu Dhabi/2021 (3)	98%	98%		97%	98%
OP324808/UAE/Abu Dhabi/2021 (4)	97%	98%	97%		98%
OM867572.1/UAE/Dubai/2022	99%	98%	98%	98%	

**Table 4:** Estimation of pairwise identity between PPRV F gene of this study strains and the newly published sequence from Barberry sheep (OM867572.1/UAE/Dubai/2022). Numbers in brackets are PPRV UAE 2021 strains.

Strain	OP324801	OP324802	OP324803	OP324804	OM867572.1
OP324801/UAE/Abu Dhabi/2021 (1)		99%	100%	100%	99%
OP324802/UAE/Abu Dhabi/2021 (2)	99%		99%	99%	98%
OP324803/UAE/Abu Dhabi/2021 (3)	100%	99%		100%	99%
OP324804/UAE/Abu Dhabi/2021 (4)	100%	99%	100%		99%
OM867572.1/UAE/Dubai/2022	99%	98%	99%	99%	

## Data Availability

The partial N and F gene sequences generated in this study are available in the NCBI database under accession numbers mentioned in the manuscript.
